# The role of social networks in the development of overweight and obesity among adults: a scoping review

**DOI:** 10.1186/s12889-015-2314-0

**Published:** 2015-09-30

**Authors:** Katie Powell, John Wilcox, Angie Clonan, Paul Bissell, Louise Preston, Marian Peacock, Michelle Holdsworth

**Affiliations:** School of Health and Related Research (ScHARR), University of Sheffield, 30 Regent Street, Sheffield, S1 4DA UK; Public Health, Nottingham City Council, Nottingham, UK

**Keywords:** Social networks, Obesity, Overweight, Contagion, Homophily, Social processes, Social capital

## Abstract

**Background:**

Although it is increasingly acknowledged that social networks are important to our understanding ofoverweight and obesity, there is limited understanding about the processes by which such networks shapetheir progression. This paper reports the findings of a scoping review of the literature that sought to identify the key processes through which social networks are understood to influence the development of overweight and obesity.

**Methods:**

A scoping review was conducted. Forty five papers were included in the final review, the findings of which were synthesised to provide an overview of the main processes through which networks have been understood to influence the development of overweight and obesity.

**Results:**

Included papers addressed a wide range of research questions framed around six types of networks: a paired network (one’s spouse or intimate partner); friends and family (including work colleagues and people within social clubs); ephemeral networks in shared public spaces (such as fellow shoppers in a supermarket or diners in a restaurant); people living within the same geographical region; peers (including co-workers, fellow students, fellow participants in a weight loss programme); and cultural groups (often related toethnicity). As individuals are embedded in many of these different types of social networks at any one time, the pathways of influence from social networks to the development of patterns of overweight and obesity are likely to be complex and interrelated. Included papers addressed a diverse set of issues: body weight trends over time; body size norms or preferences; weight loss and management; physical activity patterns; and dietary patterns.

**Discussion:**

Three inter-related processes were identified: *social contagion* (whereby the network in which people are embedded influences their weight or weight influencing behaviours), *social capital* (whereby sense of belonging and social support influence weight or weight influencing behaviours), and *social selection* (whereby a person’s network might develop according to his or her weight).

**Conclusions:**

The findings have important implications for understanding about methods to target the spread of obesity, indicating that much greater attention needs to be paid to the social context in which people make decisions about their weight and weight influencing behaviours.

**Electronic supplementary material:**

The online version of this article (doi:10.1186/s12889-015-2314-0) contains supplementary material, which is available to authorized users.

## Background

The social science literature increasingly suggests that social networks are important in the maintenance of general health and protection from a range of diseases [1]. A social network may be defined as a structure of “reciprocally oriented and dependent people” [2]. People are embedded within numerous networks of interdependency of many kinds at any one time that might reflect (among many things) kinship ties, professional connections, or place of residence. Significantly however, networks can extend beyond personal connections and include interdependencies which extend across vast geographical areas and to large numbers of people – such as those relating to the maintenance of cultural identity. Over the last 10 years, a growing body of work has focused on the role that such networks might play in understanding the development of overweight and obesity [3]. Many studies have explored the risk of becoming overweight or obese according to the size [4] and density [5] of people’s social networks. Most research attention in this field has been focussed on the characteristics of people within a particular social network (such as a friendship group) and the risk of overweight or obesity among network members, which has indicated that obesity might cluster within particular social networks [3, 6–10]. Although there is increasing acceptance that social networks are important to our understanding of obesity [8], there is as yet limited understanding about the ways in which such networks might shape the development of overweight and obesity.

Examining the types of networks that have most influence on one’s risk of overweight and obesity has prompted several different explanations for the apparent social clustering of overweight and obesity. There is evidence to suggest, for example, that obesity is more likely to cluster in same-sex friendship groups [3] but that the geographical proximity of networks has limited influence [3, 11], prompting the suggestion that social norms established through friendship groups might play an important role in the development of overweight and obesity [3]. Such explanations are, however, are disputed [9]. There has also been considerable recent interest in the social resources embedded within social networks that might affect obesity [12] Bourdieu defined social capital as “the aggregate of the actual or potential resources which are linked to possession of a durable network of more or less institutionalized relationships of mutual acquaintance and recognition” [13]. Within the field of public health, research has sought to explore a range of different forms of social capital that might influence health. Although there are variations in terminology the range covers people’s sense of belonging in particular networks [14], the practical and emotional support that people might access through networks [15], the behavioural norms that might be established through networks [16] and the development of trust or co-operation [17]. There is limited understanding about the ways in which such resources influence the development of overweight and obesity or indeed whether other social mechanisms explain its development more adequately [8].

Interventions seeking to manipulate social networks by targeting well-connected individuals might be effective in influencing weight [6] but revealed little about the processes through which they might be effective. A systematic review comparing the effectiveness of group-based to individual-based modes of treatment for adult obesity suggests that weight loss interventions are more effective when delivered in a group setting, although the pathways through which effectiveness is improved are not clear [18]. There is some indication that family-based social networking sites can influence weight influencing attitudes by exposing people in the same social network to the same information [19]. Encouraging participation in health enhancing activities might also be more successful when dense networks of friends [20] or more homogenous networks [21] are targeted as opposed to unconnected individuals. Furthermore, evidence from a randomized-controlled trial indicates that spouses may benefit from their partners’ participation in lifestyle interventions [22], suggesting that weight management interventions might create a ripple effect within social networks.

In essence, although there has been a large amount of research into social networks and weight status since 2007, the field remains under-theorised, limiting the development of explanations for the clustering of overweight and obesity within particular social networks. This paper seeks to address this gap in knowledge by examining the social processes through which social networks influence the development of overweight and obesity. Through a scoping review of the literature, this paper provides an overview of the ways in which social networks and their relationship to overweight and obesity have been understood. It draws on a sociological understanding of social processes as the pattern of events that occur out of the connections between people [23] to explain how networks might facilitate the development of overweight and obesity.

## Methods

A scoping review was conducted in order to identify and analyse the literature relating to social networks and the development of overweight and obesity. Scoping reviews are frequently used in healthcare research as a means to quickly assess the extent and range of literature in a given topic [24], making them particularly suitable for examining published work in an emerging field [25]. Such reviews are typically used to enhance understanding of the concepts, methods and approaches used to examine a particular research issue [26]. In this respect, they can be particularly useful for developing ‘conceptual clarity’ [27] in a field such as this, where social processes have been under-theorised. A number of methods were utilised to enhance the validity of the review, including the use of multiple researchers in the selection and analysis of papers, and the development of clear inclusion and exclusion criteria, which were developed iteratively and applied retrospectively to potential included references. The research team comprised five researchers, one information specialist and a public health manager in the National Health Service.

### Search process

The literature searching process was carried out in two stages. The first stage of the search focussed on a key publication by Christakis and Fowler [3], which had been identified as influential in the field of social networks and overweight/obesity due to its high citation rate over a short time period. This approach of using an influential paper as a starting point for conducting reviews is recommended by Booth et al. [28]. A database search was conducted to identify any academic papers citing this work. This search returned 490 results, which, given the short time scale since the paper’s publication, underscored its influence in the field. The abstracts of these papers were reviewed by two independent reviewers in order to develop an understanding of the literature in this area. The researchers made a note of key ideas and themes explored in these papers to inform the development of research questions for the review. This process also enabled the researchers to harvest key terms around which the second stage of the search process could be designed.

The second stage of the search process comprised a full-scale search of major health and social science databases (Medline via OVID SP; Embase via OVID SP; PsycINFO via OVID SP; AMED via OVID SP; Social Policy and Practice via OVID SP; CINAHL via EBSCO; Science Citation Index and Social Science Citation Index via Web of Knowledge; ASSIA via PROQUEST; Cochrane Database of Systematic Review via Cochrane Library; Database of Abstracts of Reviews of Effects via Cochrane Library; Cochrane Central Register of Controlled Trials via Cochrane Library; NHS EED via Cochrane Library; Econlit via OVID SP; Scopus via Elsevier; OpenGrey/Grey Nets/Google Scholar). The key terms derived from stage one of the search were combined with those included in the research protocol for this project (produced by the lead researcher of this project with expertise in the field) and terms used in a previous study for the National Institute for Research and Clinical Excellence into weight management [29]. The search was restricted to publications produced after 2002 on the basis that a shift in understanding about the causes of overweight and obesity had taken place at this time. Full details of search terms are included in the additional information (Additional file 1).

**Fig. 1:**
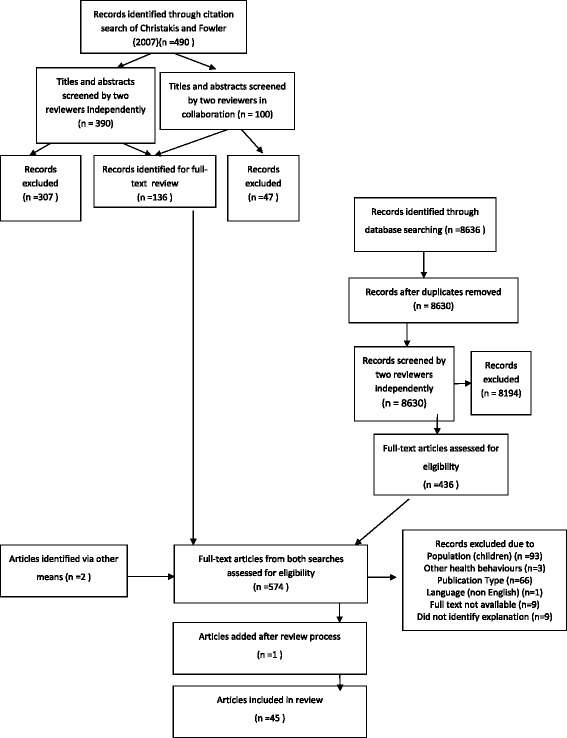
The flow of studies through the review process

The second stage of the search returned 8636 results. Search results were screened to identify papers relevant to the review. The flow of studies through the review process is shown in Fig. 1. First, the titles and abstracts of all papers were divided between two researchers and reviewed independently. Broad exclusion criteria were developed during the first part of the search process, to decisions about whether to include papers: non-human subjects; biological focus; initiative/intervention at the individual level; primary focus is another disease; focus is prevalence of obesity in particular community but not exploring explanations; explores prevalence of obesity according to individual characteristics (age/sex) rather than relational characteristics (marriage status); identifying individual characteristics associated with physical activity levels/diet; non-obesity related intervention.

The two reviewers maintained a dialogue throughout this process and the exclusion criteria were expanded as the reviewers became more familiar with the scope of the literature as Arksey and O’Malley [26] recommend. Caution was applied in the early stages of this process and any papers deemed potentially relevant were included to improve the likelihood that relevant papers were not discarded. To improve inter-reviewer reliability, spot checks were carried out on discarded references by a third reviewer.

As the researchers became more familiar with the range and extent of the literature, it became possible to define inclusion criteria, which were then applied retrospectively to papers initially identified as relevant. Following the screening of papers by title and abstract, 574 full papers were retrieved for review. The screening of full papers mirrored the screening process for titles and abstracts. One paper (published during the production of this paper) was identified after the review was completed and another two papers were identified by a colleague working in this field. For the purposes of this review, social networks were defined as networks reflecting social relations between two or more people, occurring through face-to-face or virtual interaction over any period of time. Examining the influence of organisational and institutional networks (such as the media) was considered beyond the scope of this study. Research focussed solely on children and young people was excluded partly due to time constraints and partly because the review steering group perceived that the mechanisms shaping the development of overweight and obesity among children and young people, and the role of social networks in this process, were likely to differ to those amongst adults. Adults were defined for the purposes of the review as aged 18 years or over. If the age of research participants was not made explicit in a potentially relevant paper, a screening decision was made on the basis of the author(s)’ description of the research population. Research focussed on adults and children/young people, and research following children into adulthood was included.

The final inclusion criteria developed for the review were for studies:

I. Presenting findings from analysis of primary or secondary data
II. Based on data related to adults
III. Providing an explanation for the clustering of overweight/obesity within social networks
IV. Providing an explanation for the ways in which social networks shape or are shaped by weight gain or weight loss
V. Providing an explanation for how social networks shape or are shaped by weight influencing behaviours (physical activity, diet and body size norms/preferences)

Included papers were reviewed to identify processes that might explain the development of overweight and obesity through social networks. After extracting a summary of the findings relevant to our research question, themes were developed to organise the extracted data using the framework approach for thematic analysis [30]. During the first iteration of theme development, labels for themes reflected the language used within the papers. As the researchers became more familiar with the data, themes became more abstracted, reflecting the researchers’ knowledge of this field. The search terms developed (shown in Appendix A) were used as sensitising concepts [31] in the development of appropriate theme labels that best fit the range of ideas across the data.

## Results

Forty five papers were included in the final review. The majority of studies used primary data collection methods and most of these used a quantitative approach (Table 1). Almost two-thirds of papers [29] were based on studies in North America; nine were based in Europe; two were based in Australia; one was based in Asia; one had a global reach and in three reviews the geographical location of populations was unspecified (Tables 2, 3, 4). Full details of data extraction of the selected studies can be found in are included in the additional information (Additional file 2).

**Table 1:** Methodology used in the included studies

Research strategy	Total	Longitudinal design	Cross-sectional design	Experimental design	Literature review
Quantitative	33	9	14	10	0
Qualitative	7	0	7	0	0
Mixed methods	2	0	2	0	0
N/A	3	N/A	N/A	N/A	3
Total	45	9	23	10	3

**Table 2:** Summary of ‘social contagion’ studies

Ref list #	Author	Network type	Sample	Study design	Process
[35]	Burger, J. M. 2010	Peers	USA	Experimental	Mirroring
			Female		
[37]	Carrell, S. 2011	Peers	USA	Experimental	Mirroring
			Male and female		
[39]	Lemon, S. C. 2009	Peers	USA	Quasi-experimental	Mirroring
			Male and female		
[34]	Mcferran, B. 2010	Peers	USA	Experimental	Mirroring
			Female		
[40]	Robinson, E. 2011	Peers	UK	Experimental	Mirroring
			Female		
[38]	Ali, H. I. 2010	Friends and family, cultural group	United Arab	Cross-sectional qualitative focus groups	Mirroring Social support
			Emirates		
			Females		
[32]	Befort, C. 2008	Friends and family, cultural group	USA	Qualitative focus groups	Mirroring
			Female		
[33]	Bertoni, A. 2011	Friends and family	USA	Qualitative focus groups	Mirroring
			Male and female		
[41]	Kouvonen, A. 2012	Friends and family	England	Longitudinal survey	Mirroring
			Male and female		
[36]	Pachucki, M. 2011	Friends and family	USA	Longitudinal survey	Mirroring
			Male and female		
[10]	Hruschka, D. 2011	Friends and family	USA	Cross-sectional survey	Mirroring Aspiring
			Female		
[45]	Blanchflower, D. 2009	People living in same region	Europe	Cross-sectional survey	Aspiring
			Male and female		
[44]	Bramble, J. 2009	Cultural group	USA	Qualitative interviews	Aspiring
			Female		
[43]	Renzaho, A. M. N. 2012.	Cultural group	Australia	Qualitative interviews	Aspiring
			Male and female		
[48]	Smith-Jackson, T. 2012	Peers	USA	Cross sectional survey/ qualitative interviews	Aspiring
			Female		
[46]	Chandler-Laney, P. 2009	Peers	USA	Longitudinal survey	Aspiring
			Female		
[47]	Klein, W. M. P. 2002.	Peers	USA	Cross sectional survey	Aspiring
			Female		
[42]	Krones, P. G. 2005	Peers	USA	Randomised-controlled trial	Aspiring
			Female		
[50]	Barthomeu, L. 2010.	Ephemeral network	European	Quasi- experimental	Changing behaviour
			Male and female		
[49]	Mcferran, B. 2010	Ephemeral network	USA	Experimental	Changing behaviour
			Female

**Table 3:** Summary of ‘social capital’ studies

Ref list #	Author	Network type	Sample	Study design	Process
[56]	Christian, H. 2011	Friends and family	Australia	Cross sectional survey	Belonging
			Male and female		
[12]	Holtgrave, D. 2006	Friends and family	USA	Cross-sectional survey	Belonging and social support
			Male and female		
[55]	Shankar, A. 2011	Friends and family	England	Longitudinal survey	Belonging
			Male and female		
[53]	Vaananen, A. 2009	Friends and family	Finland	Cross-sectional survey	Belonging
			Male and female		
[5]	Franzini, L. 2010	People living in same region	USA	Cross-sectional survey	Belonging
			Male and female		
[54]	Hystad, P. 2012	People living in same region	Canada	Cross-sectional survey	Belonging
			Male and female		
[51]	Brabec, M. 2007	People living in same region	Bolivia	Longitudinal survey	Belonging
			Male and female		
[52]	Veenstra, G. 2005	People living in same region	Canada	Cross-sectional survey	Belonging
			Male and female		
[57]	Pollard, T. M 2003.	Cultural group	UK	Cross-sectional survey	Belonging
			Male and female		
[60]	Boothe, A. 2011	Friends and family	USA	Secondary data analysis	Social support
			Female		
[62]	Daniels, J. 2006	Friends and family	USA	Qualitative	Social support
			Female		
[59]	Darlow, S. D. 2011	Friends and family	USA	Cross-sectional survey	Social support
			Male and female		
[64]	Hammond, R. A. 2010.	Friends and family	Unspecified	Literature review	Social support
[58]	Johnstone, R. 2009	Friends and family	UK	Qualitative interview study	Social support
			Male and female		
[61]	Mackert, M. 2011	Friends and family	USA	Cross-sectional, mixed methods online survey.	Social support
			Male and female		
[61]	Rohrer, J. E. 2004	Friends and family	USA	Cross sectional survey	Social support
			Female		
[66]	Verheijden, M. 2005	Friends and family	Unspecified	Literature review	Social support
[65]	Sobal, J. 2006.	Paired network	Unspecified	Literature review	Social support

**Table 4:** Summary of ‘homophily’ studies

Ref list #	Author	Network type	Sample	Study design	Process
[67]	Aruguete, M. S. 2009.	Paired network	USA	Cross-sectional survey	Homophily
			Male and female		
[70]	Averett, S. L. 2008.	Paired network	USA	Longitudinal survey	Homophily
			Male and female		
[69]	Nelson, L. D. 2005	Paired network	USA	Quasi-experimental	Homophily
			Male and female		
[4]	Apolloni, A. 2011	Friends and family	USA	Longitudinal survey	Homophily
			Male and female		
[71]	O’Malley, A. J. 2011	Friends and family	USA	Statistical modelling	Homophily
			Male and female		
[68]	Sikorskia, C. 2015	Friends and family	Germany	Cross-sectional survey	Homophily
			Male and female		
[7]	VanderWeele, T. J. 2011.	Friends and family	USA	Longitudinal survey	Homphily
			Male and female		

Included papers addressed a wide range of research questions framed around the following six types of networks (in some instances papers investigated a combination of these social network types): a paired network (one’s spouse or intimate partner); friends and family (including work colleagues and people within social clubs); ephemeral networks in shared public spaces (such as fellow shoppers in a supermarket or diners in a restaurant); people living within the same geographical region; peers (including co-workers, fellow students, fellow participants in a weight loss programme); and cultural groups (often related to ethnicity). As individuals are embedded in many of these different types of social networks at any one time, the pathways of influence from social networks to the development of patterns of overweight and obesity are likely to be complex and interrelated. Included papers addressed a diverse set of issues: body weight trends over time; body size norms or preferences; weight loss and management; physical activity patterns; and dietary patterns.

**Fig. 2:**
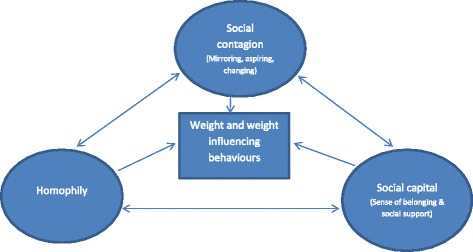
Social processes influencing the development of overweight and obesity

Three inter-related social processes emerged from the findings that explained how social networks might influence the development of overweight and obesity: *social contagion* (whereby the network in which people are embedded influences their weight or weight-influencing behaviours over time), *social capital* (whereby sense of belonging and social support influence weight or weight-influencing behaviours), and *social selection* (whereby a person’s network might develop according to his or her weight). The relationship between these categories is depicted in Fig. 2. The ways in which these processes were explained in the papers are presented in the following sections.

### Social contagion

Eleven papers identified processes through which norms and aspirations might be spread through a social network, influencing the development of overweight or obesity (Table 2). These might be defined collectively as processes of social contagion, by which the network in which people are embedded influences their weight over time. The different dimensions of contagion identified within the literature are discussed below.

#### Mirroring weight influencing behaviour of others

One of the pathways through which weight might be shaped by an individual’s social network is through mirroring the behaviour of significant others within one’s social network. This pattern was visible within friends and family networks, peer networks and cultural groups. One of the ways in which this process was explicated was through the development of eating practices in a family setting. For example, amongst obese African-American women, Befort et al. [32] found that cultural practices (such as preferences for certain foods) that were embedded within social networks influenced food choices. Within qualitative interviews, the women in this study made a strong connection between food choices and social affiliation, arguing that their eating habits bound them to their familial networks. Similarly, through qualitative focus groups, Bertoni et al. [33] observed social pressure amongst African Americans to conform to dominant food habits within family networks. Family tensions that arose when women differentiated their food practices from those of other family members were described as a barrier to making dietary changes. Other studies indicate that individuals sometimes mirror the behaviour of others with whom they have minimal social contact. In two similar experimental studies, McFerran et al. [34] and Burger et al. [35] observed that female undergraduate students in the USA base their food choices on those of others in their immediate social environment. These studies both indicated that choice of snack and snacking portion size was likely to be matched to the choices of others in a similar setting. Interestingly, Pachucki et al. [36] found that consumption of alcohol and snacks was more likely to be influenced by the consumption of similar items by others within one’s social network (friends, family and spouse) than other foods.

Similar results have been shown for physical activity norms across a range of different social settings and these patterns were particularly common within peer groups. Military trainees, for example, were likely to try and emulate the fitness levels of those in their peer groups [37]. Difficulty maintaining cultural norms within particular social settings has also been identified as a barrier to physical activity among Emirati women who perceived that walking alone in a public space for physical exercise was seen to contravene social norms [38]. A quasi-experimental study in the USA by Lemon et al. [39] indicates that workers often mirror the dietary and physical activity behaviour of their colleagues. Hospital workers who perceived that their colleagues ate healthily and undertook physical activity were more inclined to do the same. Other studies more explicitly demonstrate that mirroring might be linked to a desire to be accepted by particular groups. Indeed, Robinson et al. [40] found that desire for social acceptance may be an underlying cause of mirroring the portion size of those around us. These authors found that food mirroring between peers could be predicted by levels of self-esteem and social sense of empathy, indicating that those with less cultural competence might be more sensitive to the behaviour of others. Hruschka [10] has proposed that dietary and physical activity behaviour might be mirrored, through a process of seeking approval, even when body weight norms are not shared, but the cross-sectional study established to test this theory provided no evidence to support such an explanation. There is some indication that participating in organised clubs and organisations might reinforce bodyweight or physical activity social norms, for example Kouvonen et al. [41] found that older adults who participated in social activities were more likely to maintain a healthy body mass index (BMI) than older people who did not participate.

#### Aspiring to the body size of others in one’s social network

A range of different studies explored the ways in which body size aspirations are developed through social networks. Different types of networks have been shown to be important but peer networks seem to be particularly influential here. In a randomised controlled trial, Krones et al. [42] found that being around people that are thinner than average increased body weight dissatisfaction amongst young American women. Several studies suggest that the cultural group with which one identifies is important in shaping ideals. These cultural groups can reflect ethnicity or education level. In a qualitative cross-sectional study conducted in Australia, Renzaho [43] found that immigrant parents sought to modify their children’s weight in response to the cultural ideals of their country of origin while children sought to challenge the strategies of their parents in an attempt to conform to the perceived ideals of their Australian peers. Using survey data from North America, Hruschka [10] found evidence to indicate that friends’ body size has an influence on an individual’s body weight ideals. The study indicated that although friends do not necessarily share ideas about body size *norms*, their *ideals* may become aligned through friendship. Other studies indicate that body size norms can be established through social networks. In the USA, Bramble et al. [44] found that decisions about healthy weight were made in the context of one’s extended social network and particularly the ethnic group with which one identifies. Blanchflower et al. [45] found that perceptions about overweight among Europeans were influenced by comparisons with others and that educated individuals were more likely to rate themselves as overweight, indicating that this group aspires to a thinner ideal. Similarly, motivation for weight loss has been shown to be influenced by perceptions of the weight of one’s peers. Chandler-Laney et al. [46] showed that female participants on a weight loss programme who perceived most women their own age to be overweight lost weight more slowly (and gained weight more quickly after the intervention) than women on the programme who perceived their peer group to be slimmer. These patterns were stronger amongst European American than African American women and seemed to be mediated by the extent to which participants consciously restricted their dietary intake, which, as the authors suggest, might indicate that women who perceive their peers to be overweight are less inclined or able to restrict their diet. Related to this, American undergraduates have been shown to calculate their risk of becoming overweight through comparison with others they identify with, defined as friends or average same-sex undergraduate peers, irrespective of absolute risk [47]. A mixed methods study in a similar setting has indicated that gaining weight upon starting university is often seen as inevitable, based on anticipation that this is the norm for people their age [48].

#### Changing behaviour in response to the body sizes in a particular setting

Comparisons with others in an ephemeral network can influence dietary choices. Amongst female undergraduates in the USA, McFerran et al. [49] found that food choices were influenced by the body weight of people present in the immediate setting and this seemed to be directly related to an individual’s comparison to others in the setting. In this quasi-experimental study, non-dieters consumed a larger quantity of snacks when served by a thinner, rather than heavier waitress, whereas dieters ate more when the waitress was heavier. Dieters were also more likely to respond to food recommendations from a heavy waitress (for healthy and unhealthy food choices) than they were to a slim waitress. Similarly, the intensity of an individual’s desire to eat has been shown to be influenced by the body weight of others in the immediate setting, with observation of an obese eater decreasing appetite [50].

### Social capital

Several papers identified ways in which social capital might influence the development of overweight and obesity (Table 3). The most commonly explored type of social capital was sense of belonging.

#### Sense of belonging

The sense of belonging that people have within particular communities seems to influence weight and weight influencing behaviours in a number of different ways, depending on the cultural setting. This is most prominent within networks of friends and family and networks of people living within the same geographical region. In a longitudinal study in an Amazonian village, increased gift giving and communal work was associated with higher BMI [51]. By contrast, in North America, Veenstra et al. [52] and Holtgrave [12] both found that involvement in community organisations and public affairs, volunteerism, informal sociability, and social trust) were protective factors in the development of overweight and obesity. This indicates that sense of belonging is important but that other forms of social capital might influence obesity. One of the ways in which sense of belonging might influence obesity is through a greater sense of the value in protecting one’s health. Amongst public sector employees in Finland, a greater sense of togetherness, trust and co-operation in the workplace has been shown to reduce risk behaviours for overweight and obesity (smoking, heavy drinking and physical inactivity [53]. Similarly, a sense of being part of one’s community has been associated with greater interest in physical activity in the USA [5] and a sense of community belonging amongst one’s neighbours made people more likely to undertake behaviour change in relation to weight influencing behaviours in Hystad and Carpiano’s [54] survey of behaviour change in Canada. Both social isolation and loneliness have been associated with a greater risk of being inactive amongst adults in the UK in a longitudinal survey of physical activity levels [55]. These findings have not been replicated in all settings: in their survey of Australian adults Christian et al. [56] found no association between BMI and neighbourliness or BMI and neighbourhood cohesion. They did however find some evidence to suggest that perceptions of community safety and the amount of graffiti are associated with BMI. Similarly, challenging the notion that social isolation might increase one’s chances of becoming obese, Pollard et al. [57] found little evidence of an association between social network size and waist circumference in the UK.

#### Social support

Social support, accessed through friends and family networks, has been linked to higher self-efficacy in online support networks for diet and physical activity interventions: social support seemed to provide structure, encouragement and purpose in relation to physical activity in a study of motivation amongst individuals with schizophrenia [58]. Similarly, in a study of undergraduate students in the USA [59], individuals’ levels of exercise could be predicted by that of their friends but only when support from such friends was rated as high. Amongst overweight women in the post-partum period in the USA, social support (and expectations about such support) were shown to influence healthy behaviours [60]. Most specifically, lack of social support was identified as a barrier to weight loss in this study. These findings were echoed in a study by Mackert et al. [61] where USA adults reported that family members could undermine their attempts to implement healthy behaviours. Through qualitative methods in a similar setting, Daniels [62] provides insight into how this might be experienced, demonstrating that family demands make it harder for women to prioritise weight loss. Similarly, a study in the USA [63] found that women on low incomes with large families who did not receive support from their parents were more likely to obese that those with smaller families who had parental help but social support has not been consistently linked to higher BMI [64]. Relatedly, based on a review of literature, Sobal [65] has argued that it is more difficult for people who are overweight to develop romantic relationships or to maintain social networks due to a lack of social support. It might be difficult to put these findings into practice, however: a review of the evidence relating to social support in weight loss interventions found inconclusive evidence to detect the influence of social support on intervention outcomes [66].

### Homophily

In comparison to processes of contagion, homophily was identified in a small number of studies (seven) as a process through which paired networks and friends and family networks might develop according to people’s weight (Table 4).

In a study examining how choice of intimate partner is influenced by weight it was found that obese undergraduate students in the USA were more likely to prefer heavier partners than their non-obese peers [67]. A study in North America found that seeking marriage can lower individuals’ BMI, indicative of the importance of weight status in the formation of intimate networks [68]. There might be a number of different reasons why body size is important in intimate partner choice. An experimental study in the USA has shown that feelings of financial scarcity might determine choices about body weight acceptability of a partner: men who felt poor in this study (denoted by access to cash and levels of savings) or hungry were more likely to consider overweight intimate partners as acceptable [69]. There is some indication that people who are normal weight might reject obese people socially [70]. This might be more common among Western, white populations: Apolloni et al. [4] have shown that obesity might lead to social isolation among Americans, but the degree of isolation was more pronounced among white Americans as compared black and Native Americans. Body size might affect network permanency as well as formation: it has been shown that people of similar BMI are less likely to dissolve existing ties and more likely to form ties [71]. A longitudinal study using sensitivity analysis has indicated that social selection only partly accounts for the social clustering of obesity, suggesting that the other processes discussed in this review are also important in understanding the development of obesity [7].

## Discussion

Although there has been increasing acceptance that social networks are important to our understanding of obesity, there has been limited understanding about the ways in which such networks shape the development of overweight and obesity. This paper sought to address this gap in knowledge by identifying and synthesising the large volume of literature that explores the complex relationship between social networks and the development of overweight and obesity. The synthesis has helped to identify the processes through which social connections influence weight influencing behaviours, body size norms and body size ideals, potentially explaining why overweight and obesity cluster in social networks. Three inter-related social processes appear to explain the role of social networks in the development of overweight and obesity, namely, *social contagion* (whereby the network in which people are embedded influences their weight over time), *social capital*, (whereby sense of belonging and social support influence weight and weight influencing behaviours), and *social selection* (whereby a person’s network might develop according to his or her weight). There is apparent uncertainty in the literature as to whether processes of contagion or social selection have more influence over the clustering of overweight and obesity. Research into processes of obesity development among children has explored the interplay of these processes using a stochastic actor-based model for the co-evolution of social selection and peer influence [72], but this review indicates that application of this model to the adult population is lacking. The review indicates, however, that such processes are operating in conjunction with one another. As Shalizi and Thomas argue, contagion and selection processes are mutually influential, since our decisions about who we associate with are influenced by the ways in which these people shape our behaviour and vice versa [73].

This review reveals a great deal about the ways in which *different types* of networks affect overweight and obesity: processes of contagion were most common within friends and family networks, peer networks and cultural groups, supporting Christakis and Fowler’s findings. Mirroring of weight influencing behaviours was most common within friends and family networks and peer networks were most likely to foster aspirations towards the body size of others. This indicates that more homogenous networks might foster contagion. Sense of belonging operated to influence behaviours within friends and family networks and regional networks, while social support was a common feature within friends and family networks, indicating that social capital affects overweight and obesity within close networks and within networks that might not be maintained through personal ties. Homophily was most apparent within paired networks, indicating that partner choice is the most important factor influencing how networks develop according to body size. The vast majority of papers included in this review were from Europe, North America and Australia, limiting understanding about the extent to which these findings can be transferred to settings outside of these regions. Several examples within the literature indicate that identified processes (such as sense of belonging) operate differently in different regions of the world. More primary research is needed to test the applicability of these processes in non-Western settings.

These findings have important implications for public health practice. The review indicates that social networks can support norms and aspirations that might influence weight gain and this is evidenced in relation to groups known to engage less with health services and groups know to have worse rates of obesity [8, 62]. Peer support, as Christakis and Fowler [3] argue, particularly in some culturally homogenous contexts might provide a convincing means of modifying a person’s social network and influencing weight behaviour. Targeting existing peer support groups working in community (in particular participatory) settings offer useful opportunities to provide information on healthy eating and the medical advantages of maintaining a healthy body weight. Interventions could also target key community groups, for example church networks or other religious places of worship. Recent evidence suggests that taking account of social networks increases the cost-effectiveness of an intervention [74], but better understanding about the particular aspects of interventions that are cost-effective is needed. It is important to understand how such initiatives might influence health behaviour change in specific contexts.

A particular strength of this study was the fact that a scoping review enabled a large amount of literature from disparate fields to be brought together. The systematic approach to searching and reviewing the literature improved the development of clear inclusion and exclusion criteria. A disadvantage of the scoping review method of review is that no assessment of the quality of the literature can be made, as the primary aim of the review is to assess the scope, rather than the quality of literature in a particular field [24]. The review is also limited to articles printed in English, largely limiting the relevance of the findings to English-speaking countries. In limiting the scope of this review to relational networks, the review is unable to take into account the wider social and environmental context that shapes particular networks. For example, the influence of the built environment was not taken into account when understanding the ways in which social support operates. Similarly, social selection takes place within a context influenced by media relationships. Many of the studies examined in this review did not take this into account and so examination of these processes was beyond the scope of this paper. More research is needed that carefully theorises the ways in which networks are influenced by wider social and environmental conditions.

A number of gaps in understanding are apparent from the review. There were few studies of interventions to address overweight and obesity by targeting social networks; those studies that have assessed the impact of such interventions are largely inconclusive about the potential impact [66]. As obesity is more prevalent in deprived areas in high income countries, this study provides insight into how social networks may be influenced in these areas, and notably observes that there are clear differences within cultural groups. This evidence can be used locally and in areas with similar population groups to inform the development of innovative interventions that exploit social networks, and improve the quality and impact of public health interventions. There were few qualitative studies that sought to explain the pathways through which social networks influence the development of overweight and obesity. Further research is needed that seeks to understand the ways in which social networks are situated within particular political circumstances, physical places and food systems. As there is an indication that social networks are important, we need to know more about the ways in which social networks might be effectively manipulated to improve diet, body weight and physical activity norms. Given that there is partial evidence that social networks might be implicated in the spread of obesity and that they might impact on health behaviours, we need to know more about how social networks might be effectively manipulated to improve diet, body weight and physical activity norms.

## Conclusions

Although it is increasingly acknowledged that social networks are important to our understanding of overweight and obesity, there is limited understanding about how such networks shape their progression. This review has identified three interrelated social processes through which overweight and obesity might develop. The findings of this study have important implications for understanding about methods to target the spread of obesity, indicating that much greater attention needs to be paid to the social context in which people make decisions about their weight and weight influencing behaviours.

## Additional files

Additional file 1:
**Search terms used.** (PDF 184 kb) Additional file 2:
**Data extraction of all included studies.** (PDF 220 kb)
