# Supplementary material for: The role of social networks in the development of overweight and obesity among adults: a scoping review
Source: BMC Public Health. 2015 Sep 30;15:996. doi: 10.1186/s12889-015-2314-0 (PMC4589958; doi:10.1186/s12889-015-2314-0)
Supplement: Additional file 1: — Search terms used. (PDF 184 kb) [file 12889_2015_2314_MOESM1_ESM.pdf]

**Search Terms**

| | | |
|---|---|---|
| 1. | obes*.ti,ab. | Obesity Terms |
| 2. | (bodyweight or body weight).ti,ab. | |
| 3. | overweight.ti,ab. | |
| 4. | (body mass index or BMI).ti,ab. | |
| 5. | (weight gain* or weight change*).ti,ab. | |
| 6. | adipos*.ti,ab. | |
| 7. | *Obesity/ | |
| 8. | *Weight Gain/ | |
| 9. | *Overweight/ | |
| 10. | *Body Mass Index/ | |
| 11. | 1 or 2 or 3 or 4 or 5 or 6 or 7 or 8 or 9 or 10 | |
| 12. | (famil* or friend* or neighbour* or neighbor* or spous* or sibling* or partner* or communit* or generation*).ti,ab. | People Terms |
| 13. | (peer* or (person adj to adj person) or (face adj to adj face)).ti,ab. | |
| 14. | 12 or 13 | |
| 15. | (cluster* or contagio* or epidemi* or infect* or percepti* or spread* or rippl* or diffus*).ti,ab. | Cluster Terms |
| 16. | 14 and 15 | |
| 17. | (network* or social dynamic* or social interaction* or social tie* or social norm* or community norm* or social comparison* or social capital or social stress* or social transgression).ti,ab. | Social Network Terms |
| 18. | 11 and 16 | |
| 19. | 11 and 17 | |
| 20. | 18 or 19 | |
| 21. | limit 20 to (English language and humans and yr="2002 - 2012") | |
