# Supplementary material for: The role of social networks in the development of overweight and obesity among adults: a scoping review
Source: BMC Public Health. 2015 Sep 30;15:996. doi: 10.1186/s12889-015-2314-0 (PMC4589958; doi:10.1186/s12889-015-2314-0)
Supplement: Additional file 2: — Data extraction of all included studies. (PDF 220 kb) [file 12889_2015_2314_MOESM2_ESM.pdf]

Data extraction of all included studies

Ref list #	First author & publication date	Research questions	Network type	Sample characteristics (Region, gender, age range, ethnicity, SES, occupation, health status – where reported)	Continent	Study design & methods	Findings	Process
35	Burger, J. M. 2010	What snack choices do women make when told about the snack choices of others in similar setting?	Peers	USA<br>Female<br>18-22yrs<br>Higher education undergraduates	North America	Experimental	Women make food choices based on a descriptive norm – what they are led to believe were the food choices of other women in a similar situation	Mirroring
37	Carrell, S. 2011	Does being assigned to peer groups which are less fit during high school affect college fitness scores?	Peers	USA<br>Male and female<br>Air force Academy trainees	North America	Experimental	People mimic the diet or exercise habits of least fit friends or use their fitness as a benchmark for their own fitness levels	Mirroring
39	Lemon, S. C. 2009	Are perceptions of organizational commitment to employee health, co-worker physical activity and eating behaviours associated with BMI, physical activity and eating behaviours?	Peers	USA<br>Male and female<br>70% White, 14% Black, 12% Black, 4% Asian<br>Hospital workers	North America	Quasi-experimental	Perceptions of co-worker normative behaviours were associated with a participant's own physical activity and eating behaviours	Mirroring

Ref list #	First author & publication date	Research questions	Network type	Sample characteristics (Region, gender, age range, ethnicity, SES, occupation, health status – where reported)	Continent	Study design & methods	Findings	Process
34	Mcferran, B. 2010	Do people use the behaviour of others in shaping their own snacking consumption decisions?	Peers	USA<br>Female<br>Higher education undergraduates	North America	Experimental	People adjust portion sizes when snacking based on what other consumers select but an adjustment occurs based on the other's body type (portion size is matched when peer is thinner and reduced when peer is overweight)	Mirroring
40	Robinson, E. (2011)	Do ingratiation strategies explain social matching of food intake in young females?	Peers	UK<br>Female<br>Mean 20yrs<br>Higher Education undergraduates	Europe	Experimental	Desire for social acceptance may be an underlying cause of social matching of food intake	Mirroring
38	Ali, H. I. 2010	What are the weight management behaviours and perceptions of women who are at increased risk for type 2 diabetes?	Friends and family, cultural group	United Arab Emirates<br>Females<br>20–60yrs Considered high risk for type 2 diabetes	Asia	Cross-sectional qualitative focus groups	Weight-related behaviours affected by cultural norms – such as overeating at social engagements and perceived unacceptability of some physical activity alone<br>Competing demands (childcare and housework) was a barrier to physical activity	Mirroring<br>Social support

Ref list #	First author & publication date	Research questions	Network type	Sample characteristics (Region, gender, age range, ethnicity, SES, occupation, health status – where reported)	Continent	Study design & methods	Findings	Process
32	Befort, C. 2008	What are Obese African American women's perceptions and beliefs about body size, weight, and weight loss	Friends and family, cultural group	USA<br>Female<br>18+yrs<br>African- American<br>Obese	North America	Qualitative focus groups	Desire to conform to traditional cultural practices influences food choice	Mirroring
33	Bertoni, A. 2011	What are the perceptions of perceptions of African Americans to Dietary Approaches to Stop Hypertension (DASH) and the food environment?	Friends and family	USA<br>Male and female<br>34-63yrs<br>African- American<br>High blood pressure<br>Living low-income communities	North America	Qualitative focus groups	Social pressure to conform to established eating habits from within family network	Mirroring
41	Kouvonen, A. 2012	Is there an association between participation in social activities and weight loss?	Friends and family	England<br>Male and female<br>50+yrs	Europe	Longitudinal survey	Older adults who participated in social activities were more likely to maintain a healthy BMI than older people who did not participate	Mirroring
36	Pachucki, M. 2011	Are eating behaviours concordant among diverse sets of social ties?	Friends and family	USA<br>Male and female<br>29-85yrs	North America	Longitudinal survey	Consumption of alcohol and snacks was more likely to be influenced by the consumption of similar items by others within one's social network than other foods	Mirroring
10	Hruschka, D. 2011	Do shared body size norms play a role in the social contagion of body size and obesity?	Friends and family	USA<br>Female<br>18-45yrs	North America	Cross-sectional survey	A network member's BMI may influence an ego's desired body size, which in turn influences an ego's BMI.	Mirroring<br>Aspiring

Ref list #	First author & publication date	Research questions	Network type	Sample characteristics (Region, gender, age range, ethnicity, SES, occupation, health status – where reported)	Continent	Study design & methods	Findings	Process
45	Blanchflower, D. 2009	What is the influence of relative weight on one's desired weight?	People living within the same geographical region	Europe<br>Male and female	Europe	Cross-sectional survey	Comparisons and relative-weight concerns influence BMI	Aspiring
44	Bramble, J. 2009	Examine the cultural context for notions of good health and health practices, and perception of obesity and weight management	Cultural group	USA<br>Female<br>40-62yrs<br>African-American and African-Caribbean	North America	Qualitative interviews	Decisions about healthy weight made in the context of one's extended social network (the 'cultural' group one associates with)	Aspiring
43	Renzaho, A. M. N. 2012.	Are there intergenerational differences in food, physical activity, and body size perceptions among refugees and migrants in Australia?	Cultural group	Australia<br>Male and female<br>(Adult group – mean age 36yrs)<br>Refugees and migrants from North East Africa	Australia	Qualitative interviews	Parents aspire to influence the weight of their children according to particular cultural ideals	Aspiring
48	Smith-Jackson, T. 2012	How do people account for weight gain when starting university?	Peers	USA<br>Female<br>18-19yrs<br>Higher education undergraduates	North America	Cross sectional survey and qualitative interviews	Gaining weight upon starting university is often seen as inevitable and comparisons to others can influence weight gain	Aspiring
46	Chandler-Laney, P. 2009	Do European American and African American women take longer to lose weight and are they less likely to maintain weight loss if they perceive others to be overweight?	Peers	USA<br>Female<br>22-46yrs	North America	Longitudinal survey	Perception of peers' weight influences motivation for weight loss	Aspiring

Ref list #	First author & publication date	Research questions	Network type	Sample characteristics (Region, gender, age range, ethnicity, SES, occupation, health status – where reported)	Continent	Study design & methods	Findings	Process
47	Klein, W. M. P. 2002.	Do comparative risk estimates relative to the average peer predict dietary intentions and concern about absolute risk of overweight and obesity?	Peers	USA<br>Female<br>Higher education undergraduates	North America	Cross sectional survey	Weight comparisons with perceived average peer can shape perceived risk of obesity and dietary intention	Aspiring
42	Krones, P. G. 2005	Does social comparison with attractive peers in the real world increase body dissatisfaction and negative affect	Peers	USA<br>Female<br>17-22yrs<br>Ethnically diverse group	North America	Randomised-controlled trial	Being around thinner than average people increases dissatisfaction with body weight	Aspiring
50	Barthomeu, L. 2010.	Is eating desire lower in the presence of an obese rather than a normal-weight eater and is this affected by facial expression and one's own weight status?	Ephemeral network	European<br>Male and female<br>19- 37yrs	Europe	Quasi-experimental	Desire to eat affected by others' facial responses and their weight status	Changing behaviour
49	Mcferran, B. 2010	Does the body type of others in vicinity affect food consumption?	Ephemeral network	USA<br>Female<br>Higher education undergraduates	North America	Experimental	Dietary choices influenced by body weight of people present in the choice setting	Changing behaviour

Ref list #	First author & publication date	Research questions	Network type	Sample characteristics (Region, gender, age range, ethnicity, SES, occupation, health status – where reported)	Continent	Study design & methods	Findings	Process
56	Christian, H. 2011	What are the individual, behavioural, social and built environment correlates of BMI?	Friends and family	Australia<br>Male and female<br>Mean age 40yrs	Australia	Cross sectional survey	Perceived graffiti and vandalism was positively associated with BMI, and participants who perceived that their neighbourhood was safer from crime and safer for pedestrians had a lower BMI. Perceived neighbourhood cohesion and neighbourliness not associated with BMI.	Belonging
12	Holtgrave, D. 2006	Is social capital a protective factor against obesity and diabetes?	Friends and family	USA<br>Male and female	North America	Cross-sectional survey	Community organizational life, involvement in public affairs,<br>volunteerism, informal sociability, and social trust – a combined high score is a protective factor in development of obesity	Belonging and social support
55	Shankar, A. 2011	What impact does social isolation and loneliness have on health-related behaviour?	Friends and family	England<br>Male and female<br>50yrs+<br>30% with long-term limiting health condition	Europe	Longitudinal survey	Physical activity habits shaped by extent of social contact with others in social clubs	Belonging
53	Vaananen, A. 2009	Is there an association between social cap and CVD risk factors?	Friends and family	Finland<br>Male and female<br>Public sector employees	Europe	Cross-sectional survey	Increased workplace social capital (sense of togetherness, trust and co-operation) is associated with fewer health risk behaviours	Belonging

Ref list #	First author & publication date	Research questions	Network type	Sample characteristics (Region, gender, age range, ethnicity, SES, occupation, health status – where reported)	Continent	Study design & methods	Findings	Process
5	Franzini, L. 2010	Which socio-economic and ethnic disparities in neighbourhood characteristics are associated with outdoor physical activity?	People living within the same geographical region	USA<br>Male and female<br>Caregivers to primary-school-aged children	North America	Cross-sectional survey	Being part of the community associated with increased interest in physical activity	Belonging
54	Hystad, P. 2012	Is there an association between sense of belonging and likelihood of undertaking behaviour change?	People living within the same geographical region	Canada<br>Male and female<br>12+yrs	North America	Cross-sectional survey	The ways in which people relate to others in their geographical community might influence the way in which they feel about health/weight management	Belonging
51	Brabec, M. 2007	What is the relationship between BMI, relative income, relative social capital, village income, and village social capital?	People living within the same geographical region	Bolivia<br>Male and female<br>Head of household<br>Tsimane people	South America	Longitudinal survey	Comparisons with others in one's community influences BMI	Belonging
52	Veenstra, G. 2005	To what degree are relationships between social capital and health embedded in local geographical contexts and influenced by demographic factors, socio-economic status, health behaviours and coping skills?	People living within the same geographical region	Canada<br>Male and female<br>Mean 46yrs	North America	Cross-sectional survey	Participation in social groups is associated with lower body weight	Belonging

Ref list #	First author & publication date	Research questions	Network type	Sample characteristics (Region, gender, age range, ethnicity, SES, occupation, health status – where reported)	Continent	Study design & methods	Findings	Process
57	Pollard, T. M 2003.	Does social isolation contribute to high rates of CHD in the South Asian UK population?	Cultural group	UK<br>Male and female<br>25-74yrs<br>Indian, Pakistani and Bangladeshi and European origin	Europe	Cross-sectional survey	Social network size and waist size might not be related	Belonging
60	Boothe, A. 2011.	What are the predictors of unmet social support for healthy behaviours?	Friends and family	USA<br>Female<br>18+yrs<br>Overweight<br>Post-partum period	North America	Secondary data analysis of trial data	Social support (and expectations about such support) influences healthy behaviours.	Social support
62	Daniels, J. 2006.	What are the experiences of weight loss among women?	Friends and family	USA<br>Female<br>32-55yrs<br>Trying to lose weight without surgery or medication	North America	Qualitative	Family demands make it harder to prioritise weight loss	Social support
59	Darlow, S. D. 2011	What is the influence of perceived support for exercise as well as close others' exercise habits on own exercise?	Friends and family	USA<br>Male and female<br>18-26yrs<br>Higher education undergraduates	North America	Cross-sectional survey	Exercise habits of close others are associated with one's own exercise habits when those others offer support.	Social support
64	Hammond, R. A. 2010.	What is the role of social influence in the obesity epidemic?	Friends and family	Unspecified	Unspecified	Literature review	Stress and social support not related to BMI	Social support
58	Johnstone, R. 2009.	What are the barriers to uptake of and adherence to physical activity in community dwelling patients with a diagnosis of schizophrenia?	Friends and family	UK<br>Male and female<br>18-60 yrs<br>People with Schizophrenia	Europe	Qualitative interview study	Social support influences exercise habits	Social support

Ref list #	First author & publication date	Research questions	Network type	Sample characteristics (Region, gender, age range, ethnicity, SES, occupation, health status – where reported)	Continent	Study design & methods	Findings	Process
61	Mackert, M. 2011	Does social undermining influence health behaviours?	Friends and family	USA<br>Male and female<br>Mean age 43yrs	North America	Cross-sectional, online survey. Qualitative and quantitative component	Undermining affects health behaviour change	Social support
61	Rohrer, J. E. 2004	Is there an association between social support and obesity prevalence?	Friends and family	USA<br>Female<br>Low income<br>48% obese	North America	Cross sectional survey	Family responsibilities might be associated with obesity	Social support
66	Verheijden, M. 2005.	What role does social support play in weight loss interventions?	Friends and family	Unspecified	Unspecified	Literature review	Social support might not be related to weight intervention outcomes	Social support
65	Sobal, J. 2006..	How does being overweight impact on social relationships?	Paired network	Unspecified	Global	Literature review	People who are overweight might have less social support	Social support
67	Aruguete, M. S. 2009.	How is choice of romantic partner influenced by weight?	Paired network	USA<br>Male and female<br>Undergraduate students	North America	Cross-sectional survey	Obese people prefer heavier partners	Homophily
70	Averett, S. L. 2008.	Is there an association between relationship status and BMI?	Paired network	USA<br>Male and female<br>14-22 yrs	North America	Longitudinal survey	Seeking marriage can lower BMI, social obligations that develop through being in a relationship can increase BMI	Homophily
69	Nelson, L. D. 2005	Do situational feelings of resource scarcity predict personal preferences for prospective partner's body weight?	Paired network	USA<br>Male and female<br>Higher education undergraduates	North America	Quasi-experimental	How one sees oneself in relation to others determines choices about body weight acceptability of partner	Homophily

Ref list #	First author & publication date	Research questions	Network type	Sample characteristics (Region, gender, age range, ethnicity, SES, occupation, health status – where reported)	Continent	Study design & methods	Findings	Process
4	Apolloni, A. 2011	Do marginalised children and young people remain marginalised in adulthood? Does obesity precede marginalisation or vice versa?	Friends and family	USA<br>Male and female<br>Young adults<br>Ethnically diverse population	North America	Longitudinal survey	Obesity precedes social isolation – particularly White Americans	Homophily
71	O'Malley, A. J. 2011	What effect do health traits have on changes in friendship ties?	Friends and family	USA<br>Male and female	North America	Statistical modelling	People of similar BMI are less likely to dissolve existing ties	Homophily
68	Sikorskia, C. 2015	To what extent does BMI influence social distancing?	Friends and family	Germany<br>Male and female	Europe	Cross-sectional survey	Seeing marriage lowers BMI	Homophily
7	VanderWeele, T. J. 2011.	To what extent might supposed contagion effects for obesity, smoking, happiness, and loneliness be explained away by homophily or confounding?	Friends and family	USA<br>Male and female	North America	Longitudinal survey	Social selection only partly accounts for the social clustering of obesity	Homphily
